# Re-burying Artificially Exposed Surface of Viral Subunit Vaccines Through Oligomerization Enhances Vaccine Efficacy

**DOI:** 10.3389/fcimb.2022.927674

**Published:** 2022-06-29

**Authors:** Xuelian Han, Zhuming Cai, Yulong Dai, He Huang, Xiangwen Cao, Yuan Wang, Yingying Fang, Gang Liu, Min Zhang, Yuhang Zhang, Binhui Yang, Wei Xue, Guangyu Zhao, Wanbo Tai, Min Li

**Affiliations:** ^1^ State Key Laboratory of Pathogen and Biosecurity, Institute of Microbiology and Epidemiology, Academy of Military Medical Sciences, Beijing, China; ^2^ Institute of Infectious Diseases, Shenzhen Bay Laboratory, Shenzhen, China; ^3^ Institute of Hemu Biotechnology, Beijing Hemu Biotechnology Co., Ltd, Beijing, China; ^4^ Public Health School, Mudanjiang Medical University, Mudanjiang, China; ^5^ School of Basic Medical Sciences, Anhui Medical University, Hefei, China

**Keywords:** Japanese encephalitis virus, envelope protein, envelope domain III, subunit vaccines, oligomerization, vaccine design

## Abstract

Viral subunit vaccines often suffer low efficacy. We recently showed that when taken out of the context of whole virus particles, recombinant subunit vaccines contain artificially exposed surface regions that are non-neutralizing and reduce their efficacy, and thus these regions need to be re-buried in vaccine design. Here we used the envelope protein domain III (EDIII) of Japanese encephalitis virus (JEV), a subunit vaccine candidate, to further validate this important concept for subunit vaccine designs. We constructed monomeric EDIII, dimeric EDIII *via* a linear space, dimeric EDIII *via* an Fc tag, and trimeric EDIII *via* a foldon tag. Compared to monomeric EDIII or linearly linked dimeric EDIII, tightly packed EDIII oligomers *via* the Fc or foldon tag induce higher neutralizing antibody titers in mice and also protect mice more effectively from lethal JEV challenge. Structural analyses demonstrate that part of the artificially exposed surface areas on recombinant EDIII becomes re-buried in Fc or foldon-mediated oligomers. This study further establishes the artificially exposed surfaces as an intrinsic limitation of subunit vaccines, and suggests that re-burying these surfaces through tightly packed oligomerization is a convenient and effective approach to overcome this limitation.

## Significance

When recombinant viral subunit vaccines are made, large areas on their surface become artificially exposed and contain non-neutralizing regions that reduce their efficacy. To overcome this intrinsic limitation of subunit vaccine design, this study investigated whether oligomerization of subunit vaccines can re-bury part of their artificially exposed surface regions and hence enhance their efficacy. To this end, this study designed different oligomers of a Japanese encephalitis virus (JEV) subunit vaccine comprised of its envelope E protein domain III (EDIII), and revealed that when EDIII forms tightly packed oligomers, both the neutralizing immunogenicity and protective efficacy were significantly improved. Therefore, oligomerization of viral subunit vaccines is a convenient and effective approach for the design and development of highly efficacious viral subunit vaccines.

## Introduction

Compared to traditional viral vaccines such as live attenuated or inactivated virus particles, recombinant viral subunit vaccines are safe and convenient, but often suffer low efficacy (Dormitzer et al., 2008; Kwong et al., 2011; Dormitzer et al., 2012; Kulp and Schief, 2013; Wan et al., 2021; Islam et al., 2022). In a recent study, we identified an intrinsic limitation of subunit vaccine design that at least partially accounts for the low efficacy of viral subunit vaccines (Kwon et al., 2012; Wang et al., 2015; Du et al., 2016). Specifically, when recombinant subunit vaccines are taken out of the context of the whole virus particle, large surface areas on the subunit vaccines that were previously buried on the whole virus particle now become exposed. These artificially exposed surfaces may contain immunodominant non-neutralizing epitopes that can distract the immune system from reacting to neutralizing epitopes. As a result, subunit vaccines fail to induce sufficient neutralizing immune responses, and their efficacy is significantly hampered. To overcome the above intrinsic limitation of subunit vaccines, the non-neutralizing epitopes on their surface need to be covered so that they can no longer be accessible to the immune system. Using a subunit vaccine comprised of the receptor-binding domain (RBD) of the Middle East respiratory syndrome coronavirus (MERS-CoV), we showed that engineered glycan probes can mask these non-neutralizing epitopes on subunit vaccines, causing the host immune system to refocus on neutralizing epitopes and leading to enhancement of vaccine efficacy. Other than the glycan probe approach, epitope resurfacing can alter non-neutralizing epitopes and reduce their negative contribution to vaccine efficacy (Wu et al., 2010; Malito et al., 2013; Cabrera Infante et al., 2014; Zhou et al., 2020). However, both of these approaches require identification of individual immunodominant non-neutralizing epitopes, which can be plenty on the surface of subunit vaccines. A more convenient approach to generally cover non-neutralizing regions can facilitate the development of highly efficacious subunit vaccines.

Japanese encephalitis virus (JEV) is the leading cause of epidemic viral encephalitis in humans in Asian countries, with case fatality rates of ~25-30% (Morita et al., 2015; Dong and Soong, 2021; Ramli et al., 2022; Xu et al., 2022). It is a member of the Flavivirus family and belongs to the arbovirus genus; this genus also includes human pathogens Zika virus (ZIKV), West Nile virus (WNV), Dengue virus (DENV), and Yellow Fever virus (YFV) (Kuno et al., 1998; Van den Eynde et al., 2022). JEV is an enveloped and positive-stranded RNA virus. The virus-surface envelope protein (E) is responsible for JEV entry into host cells. During virus entry, the E protein of JEV first binds to a receptor on the host cell surface for viral attachment and subsequently fuses viral and host membranes, although the receptor for JEV is still being investigated (Liu et al., 2015). The tertiary structures of the E proteins from JEV and several other flaviviruses have been determined (Kanai et al., 2006; Luca et al., 2012). The flavivirus E proteins all form dimers, and the dimers further pack following a five-fold symmetry on virus surfaces (Kuhn et al., 2002). Each monomeric E protein contains four domains; among these domains, domain III (EDIII) contains the receptor-binding site and induces protective neutralizing antibodies (Mason et al., 1989; Wu et al., 2003; Alka et al., 2007). Thus, the JEV EDIII is a subunit vaccine candidate against JEV infections. However, like other viral subunit vaccines, recombinant JEV EDIII vaccine suffer low efficacy (Mason et al., 1989; Wu et al., 2003; Alka et al., 2007). Enhancing the efficacy of JEV EDIII subunit vaccines is a high priority in humans’ battle against JEV infections.

Here we show that when recombinant JEV EDIII molecules are made in tightly packed oligomeric forms, the potentially non-neutralizing regions on the artificially exposed surface of JEV EDIII can be re-buried at the oligomer interface, enhancing the vaccine efficacy. This study confirms artificially exposed surfaces as an intrinsic limitation of viral subunit vaccines, establishes oligomerization as a convenient approach to overcome this limitation, and reveals JEV EDIII in tightly packed oligomeric forms as promising subunit vaccine candidate to combat JEV infections.

## Materials and Methods

### Ethics Statement

All experiments involving animals were approved by the Institutional Animal Care and Use Committee (IACUC) of the Laboratory Animal Center, State Key Laboratory of Pathogen and Biosecurity, Beijing Institute of Microbiology and Epidemiology (Permit Number IACUC-DWZX-2021-071).

### Expression and Purification of Recombinant JEV EDIII fragments

The recombinant JEV P3 strain EDIII fragments were expressed and purified as previously described (Du et al., 2013). Briefly, JEV EDIII fragments (residues 292-402; accession number AY243844) were expressed in HEK293T cells and secreted into the cell culture medium. EDIII, EDIII-EDIII, EDIII-Fd and E with a C-terminal His_6_ tag were purified on Ni-NTA column (Qiagen), and EDIII-Fc was purified on Protein A affinity column (GE Healthcare). The yeild of used expression and purification system for each recombinant protein is larger than 0.5 mg/L, and the purity of each recombinant protein is higher than 95%.

### SDS-PAGE and Western Blot

The purified recombinant EDIII fragments were run on 10% Tris-Glycine gels, and then either stained with Coomassie blue or transferred onto nitrocellulose membranes for Western blot analysis. For Western blot analysis, after blocking with 5% non-fat milk in PBST overnight at 4°C, the membranes were incubated with an EDIII-specific mAb (1:1,000, LSBio) for 1.5 h at room temperature. After three washes, the membranes were incubated with horseradish peroxidase (HRP)-conjugated goat anti-mouse IgG (1:5,000, Invitrogen) for 0.5 h at 37°C, followed by incubation with ECL Western blot substrate reagents.

### Flow Cytometry

Binding of recombinant EDIII fragments to Vero cells was measured using flow cytometry analysis as previously described with some modifications (Du et al., 2013). Briefly, proteins (20 μg/mL) were incubated with Vero cells for 30 min at 37°C. After washes, cells were stained with FITC-labeled anti-His-tag antibodies (for proteins with His tag), or anti-human IgG-Fc antibodies (for proteins with Fc tag) for 20 min at room temperature. BSA at the same concentration was used as the control. The binding of the proteins to cells was analyzed using flow cytometer.

### Mouse Immunization and Virus Challenge

Mouse immunization and JEV challenge were carried out as previously described with modifications (Alka et al., 2007). Briefly, six- to eight-week-old female BALB/c mice were intramuscularly immunized with one of the recombinant EDIII fragments (10 μg/mouse) in the presence of aluminum adjuvant (Invitrogen), and boosted once at 4-week intervals. PBS was used as the control. Mouse sera were collected 10 days post-boost for the detection of EDIII-specific total IgG antibodies and neutralizing antibodies. Three months post-boost, mice were challenged with a lethal dose (100-Fold LD_50_) of JEV P3 strain (accession number AY243844), harvested from infected Vero cells supernatant. Survival rate and body weight of the mice were then monitored daily for 10 days.

### ELISA

The titers of JEV E-specific total IgG antibodies in mouse sera were measured using ELISA as previously described with some modifications (Wu et al., 2003). Briefly, 96-well ELISA plates were pre-coated with full-length JEV E protein (1 μg/mL, produced in Lab), and blocked with 2% non-fat milk-PBST for 2 h at 37°C. Serially diluted mouse sera were then added to the plates, and incubated for 2 h at 37°C. After four washes with PBST, E protein-bound antibodies were incubated with HRP-conjugated goat anti-mouse IgG (1:5,000, Invitrogen) for 1 h at 37°C. Then ELISA substrate 3,3’,5,5’-tetramethylbenzidine (TMB) (Invitrogen) was added. After incubation for 10 min, the reaction was stopped by 1N H_2_SO_4_. The absorbance of resulting ELISA products at 450 nm was measured using ELISA plate reader (Tecan).

### Neutralization Assay

Plaque reduction neutralization test (PRNT) was carried out to measure the titers of neutralizing antibodies in mouse sera as previously described (Verma et al., 2009). Briefly, the viral suspension of JEV (P3 strain) at 100 PFU were incubated with serially diluted sera for 90 min at 37°C. The sera-virus mixtures were transferred onto monolayers of BHK-21 cells. After incubation for 1 h at 37°C, overlay medium (1% carboxymethyl cellulose in DMEM containing 2% FBS) was added and cultured further at 37°C for 5 days. The number of plaque formation was counted after stained by 0.5% crystal violet. The titers of neutralizing antibodies are presented as the highest dilution of sera that result in a complete inhibition of virus infectivity in at least 50% of the wells (NT_50_).

### Statistical Analysis

Statistical significance among different groups was calculated by Student’s t test using GraphPad Prism statistical software. *, **, and *** indicate *P* < 0.05, *P <*0.01, and *P <*0.001, respectively.

## Results

### Design of Recombinant JEV EDIII Fragments

**Figure 1:**
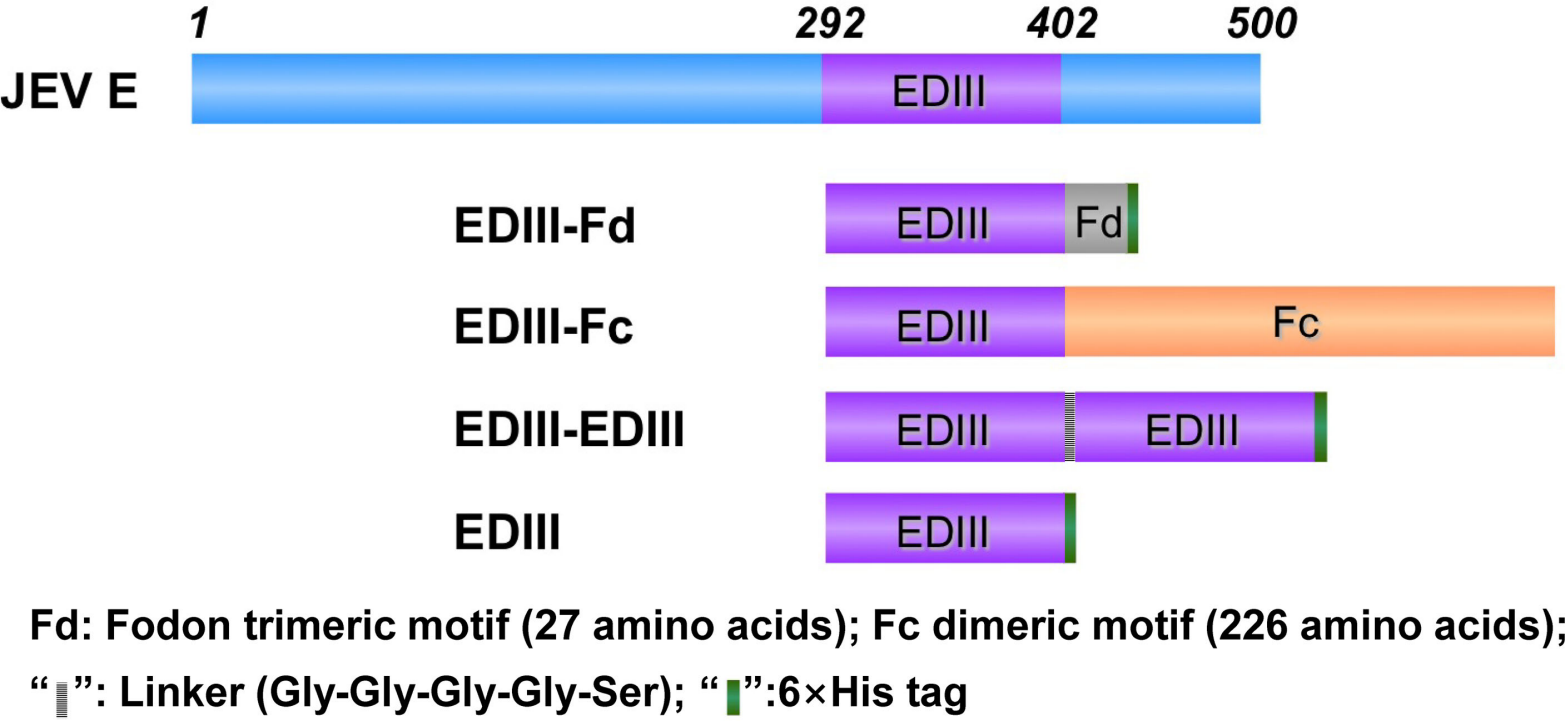
Design of recombinant JEV EDIII fragments. Schematic diagrams of JEV E protein and recombinant EDIII fragments are shown. EDIII contains a C-terminal His6 tag; EDIII-EDIII was constructed by linking two EDIII molecules head to tail through a short linear spacer, and it also contains a C-terminal His6 tag, EDIII-Fc was constructed through a C-terminal Fc dimerization tag; EDIII-Fd was constructed through a C-terminal foldon trimerization tag, and it also contains a C-terminal His6 tag.

This study aimed to investigate whether oligomerization of recombinant viral subunit vaccines can cluster vaccine molecules together and re-bury the artificially exposed surface regions of the subunit vaccines. To this end, we designed four different recombinant JEV EDIII fragments: monomeric EDIII molecule (i.e., EDIII), two EDIII molecules linked head to tail through a short linear spacer Gly-Gly-Gly-Gly-Ser (i.e., EDIII-EDIII), two EDIII molecules clustered side by side through a Fc dimerization tag (i.e., EDIII-Fc), and three EDIII molecules clustered side by side through a foldon trimerization tag (i.e., EDIII-Fd) (**Figure 1**). The goal of these designs was to produce EDIII fragments in different oligomeric forms: monomer (EDIII), loosely packed linear dimer (EDIII-EDIII), tightly packed side-by-side dimer (EDIII-Fc), and tightly packed side-by-side trimer (EDIII-Fd).

### Preparation and Biochemical Characterization of Recombinant JEV EDIII Fragments

**Figure 2:**
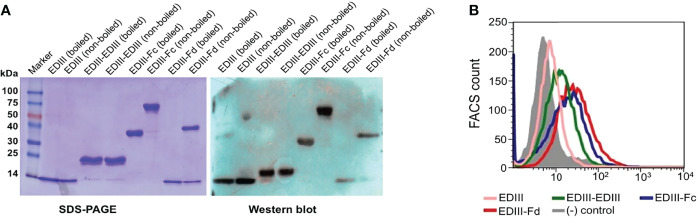
Biochemical characterization of recombinant JEV EDIII fragments. **(A)** SDS-PAGE and Western blot analyses of purified recombinant EDIII fragments. Protein samples (10 μg) were either boiled at 95°C for 5 min or not boiled, and then subjected to either SDS-PAGE and Coomassie blue staining (left) or Western blot and recognition by an EDIII-specific mAb (right). **(B)** Flow cytometry analysis of the binding of recombinant EDIII fragments to JEV-susceptible Vero cells. Cells were sequentially incubated with either one of the recombinant EDIII fragments (20 µg/mL) or BSA control (gray shade), followed by incubation with either FITC-labeled anti-human IgG (for EDIII-Fc) or anti-His tag antibody (for EDIII, EDIII-EDIII, EDIII-Fd).

We expressed and purified the above four recombinant JEV EDIII fragments as secreted proteins in mammalian cells. We performed gel electrophoresis of the purified EDIII fragments to detect their quality and oligomeric state (**Figure 2A**). After Coomassie blue staining, all of the purified EDIII fragments were shown to be homogenous. Moreover, after being boiled for complete denaturing, the EDIII fragments migrated on the gel at the same speed as their monomeric molecular weight indicated (~13 kDa for EDIII, ~25 kDa for EDIII-EDIII, ~35 kDa for EDIII-Fc, and ~13 kDa for EDIII-Fd). On the other hand, when not being boiled to partially preserve their native structure, the EDIII fragments migrated on the gel at the same speed as their oligomeric molecular weight indicated (~13 kDa for EDIII, ~25 kDa for EDIII-EDIII, ~70 kDa for EDIII-Fc, and ~40 kDa for EDIII-Fd). Thus, all of the EDIII fragments are in their respective oligomeric state as expected. We also performed Western blot of the purified EDIII fragments using a monoclonal antibody (mAb) specifically targeting JEV EDIII (**Figure 2A**). The result showed that all of the EDIII fragments bound strongly to the mAb, suggesting that the recombinant EDIII fragments maintained their antigenic structure. Furthermore, we carried out flow cytometry to investigate whether the recombinant EDIII fragments bound to JEV-susceptible cells (**Figure 2B**). The result showed that all of the EDIII fragments bound to the cells, but with different binding affinity: low affinity for EDIII, higher affinity for EDIII-EDIII, highest affinity for EDIII-Fc and EDIII-Fd. The high cell-binding affinity of oligomeric EDIII fragments likely resulted from their multivalent receptor-binding sites. Overall, the purified recombinant EDIII fragments maintain their antigenic structure, receptor-binding site, and form oligomers as designed.

### Immunogenicity of Recombinant JEV EDIII Fragments

**Figure 3:**
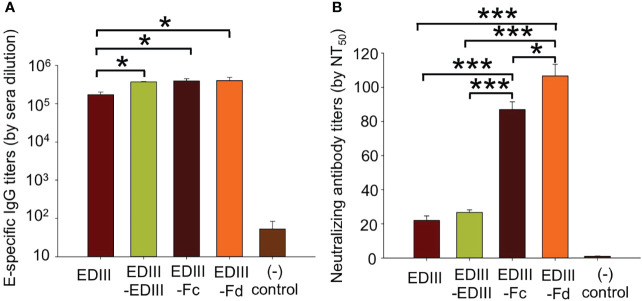
Immunogenicity of recombinant JEV EDIII fragments. **(A)** Measurement of the titers of total IgG antibodies in sera of mice immunized with one of the EDIII fragments. To this end, ELISA was carried out between the full-length JEV E protein and mouse sera. The titers of JEV E-specific total IgG antibodies are expressed as the endpoint dilutions that remain positively detectable. **(B)** Measurement of the titers of neutralizing antibodies in sera of mice immunized with one of the EDIII fragments. To this end, plaque reduction neutralization assay was performed to detect the efficiency of mouse sera in inhibiting the formation of JEV plaques. The titers of neutralizing antibodies are presented as the highest dilution of sera that result in a complete inhibition of virus infectivity in at least 50% of the wells (NT_50_). In both panels, PBS was used as a control, and the data are presented as mean ± SD of five mice in each group, *P < 0.05, ***P < 0.001.

We evaluated the immunogenicity of recombinant JEV EDIII fragments. To this end, mice were immunized with one of the JEV EDIII fragments, respectively, and their sera were subsequently analyzed for the titers of JEV E-specific total IgG antibodies and neutralizing antibodies. ELISA was performed between the full-length JEV E protein and each of the mouse sera to measure the titers of JEV E-specific total IgG antibodies induced by each of the EDIII fragments (**Figure 3A**). The results showed that all of the EDIII fragments induce the production of high titers of JEV E-specific total IgG antibodies. Moreover, plaque reduction neutralization assay was carried out to detect the efficiency of each of the mouse sera in inhibiting the formation of JEV plaques on monolayers of JEV-susceptible cells, a reflection of the titers of neutralizing antibodies in each of the mouse sera (**Figure 3B**). EDIII-Fc and EDIII-Fd are both significantly more efficient than EDIII and EDIII-EDIII in the capacity to induce the production of neutralizing antibodies. Therefore, all of the EDIII fragments are immunogenic, but EDIII-Fc and EDIII-Fd have significantly higher neutralizing immunogenicity than EDIII and EDIII-EDIII.

### Efficacy of Recombinant JEV EDIII Fragments in Protecting Animals From Lethal JEV Challenge

**Figure 4:**
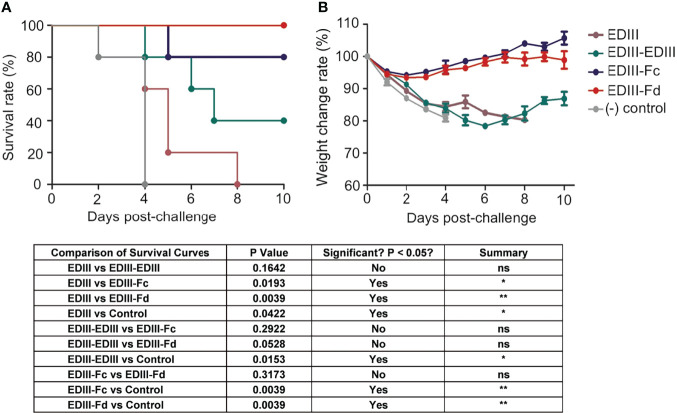
Efficacy of recombinant JEV EDIII fragments in protecting animal models from lethal JEV challenge. Mice were immunized with one of the recombinant EDIII fragments, respectively, and then challenged with JEV (P3 strain, 100× LD_50_). Challenged mice were observed daily for survival rate **(A)** and weight change rate **(B)**. The data are presented as mean ± SD of five mice in each group. *P < 0.05, **P < 0.01 and "ns" means no significance.

We investigated the efficacy of recombinant JEV EDIII fragments in protecting mice from lethal JEV challenge. To this end, mice were immunized with one of the JEV EDIII fragments, respectively, challenged with JEV, and then monitored for their survival rate and weight change rate (**Figures 4A, B**). Non-immunized mice were used as controls. The result showed that upon JEV challenge, EDIII-Fd and EDIII-Fc conferred 100% and 80% protection, respectively, for immunized mice. In contrast, EDIII-EDIII conferred only 40% protection for immunized mice, and mice immunized with EDIII or non-immunized mice all died on the 8^th^ day and 4^th^ day post-infection, respectively. Consistent with the survival rates of immunized mice, the weight change rate of mice immunized with EDIII-Fd or EDIII-Fc were much higher than those of mice immunized with EDIII, EDIII-EDIII, or non-immunized mice. Thus, EDIII-Fd and EDIII-Fc confer significantly higher protection for immunized mice from JEV infections than EDIII-EDIII and EDIII, which is consistent with the neutralizing immunogenicity of these recombinant EDIII fragments.

### Modeling Tertiary Structures of JEV EDIII Fragments

**Figure 5:**
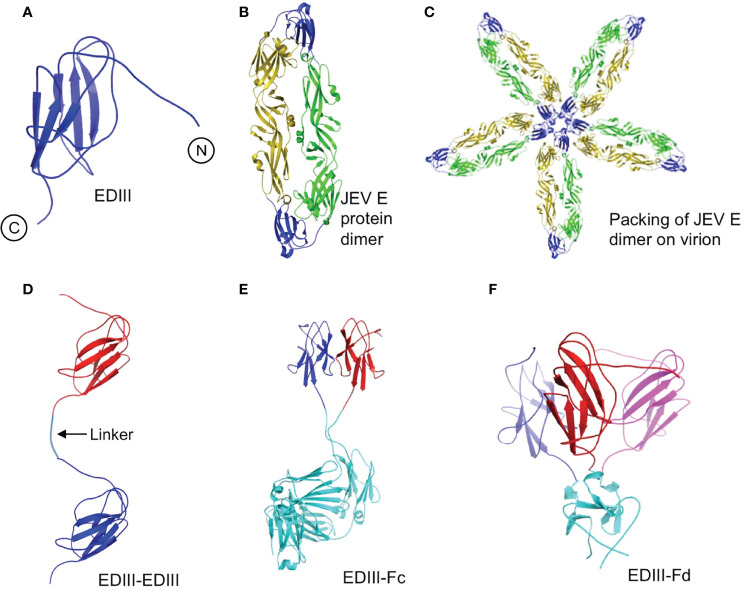
Modeled tertiary structures of JEV EDIII fragments. **(A)** EDIII of JEV E protein (PDB ID: 3P54). N- and C-terminal are shown in figure. **(B)** Dimeric JEV E protein (PDB ID: 3P54). EDIII is in blue. **(C)** Dimeric JEV E protein packed in a five-fold symmetry as on JEV virions (Kanai et al., 2006). **(D)** EDIII-EDIII. Two EDIII molecules (in blue and red, respectively) are linked head to tail through a short linear spacer (shown in the figure). **(E)** EDIII-Fc. Two EDIII molecules (in blue and red, respectively) are tightly packed side by side through an Fc dimerization tag (in cyan; PDB ID: 1IGT). **(F)** EDIII-Fd. Three EDIII molecules (in blue, red and magenta, respectively) are tightly packed side by side through a foldon trimerization tag (in cyan; PDB ID: 4NCV).

To understand the spatial relationships among different subunits in EDIII oligomers, we constructed tertiary structural models for each of the recombinant EDIII fragments (**Figure 5**). EDIII is part of the full-length dimeric E protein that further packs following a five-fold symmetry on mature JEV virus particles (**Figures 5A–C**). Thus, monomeric EDIII contains large areas of artificially exposed surface regions. Like EDIII, EDIII-EDIII also contains large exposed surface areas because there is little contact between the two subunits in EDIII-EDIII (**Figure 5D**). In contrast, the subunits in both EDIII-Fc and EDIII-Fd are tightly packed together with significant inter-subunit contacts (**Figures 5E, F**). Thus, compared with EDIII and EDIII-EDIII, both EDIII-Fc and EDIII-Fd contain much less exposed surface areas. Therefore, when taken out of the context of the JEV particles, recombinant EDIII contains artificially exposed surface areas that still exist in loosely packed EDIII dimer, but become re-buried in tightly packed EDIII dimer and trimer.

## Discussion

It had been a long-time mystery why recombinant viral subunit vaccines often suffer low efficacies. Using MERS-CoV spike protein receptor-binding domain (RBD) as a model system, our recent study revealed an intrinsic limitation associated with subunit vaccine designs (Du et al., 2016). That is, when recombinant subunit vaccines are taken out of the context of the whole virus particles, large surface areas on the subunit vaccines become artificially exposed; these artificially exposed areas contain immunodominant non-neutralizing epitopes that distract the host immune system from reacting to neutralizing epitopes and thus reduce vaccine efficacy. To overcome this intrinsic limitation, these immunodominant non-neutralizing epitopes need to be masked by a glycan probe or resurfaced. However, both the glycan probe and epitope resurfacing approaches require identification of individual non-neutralizing epitopes. Using JEV EDIII as a model system, the current study aimed to establish tightly packed oligomerization of subunit vaccines as a convenient and efficient approach to re-bury artificially exposed non-neutralizing regions and enhance the protective efficacy of viral subunit vaccines.

In this study we designed four JEV EDIII fragments: EDIII (monomeric subunit vaccine), EDIII-EDIII (linearly linked dimer), EDIII-Fc (Fc-tagged dimer), and EDIII-Fd (foldon-tagged trimer). We examined the biochemical features, immunogenicity and protective efficacy of each of these EDIII fragments. EDIII-Fc and EDIII-Fd demonstrate higher neutralizing immunogenicity and protective efficacy than EDIII and EDIII-EDIII. Previous studies also revealed good neutralizing immunogenicity and protective efficacy of Fc-tagged and Fd-tagged coronavirus subunit vaccines (Ma et al., 2014; Tai et al., 2016; Du et al., 2017; Tai et al., 2017). However, different from these previous studies, the current study performed detailed comparisons among different JEV EDIII fragments including monomer, linearly linked dimer, Fc-tagged dimer, and Fd-tagged trimer. Our results suggest that compared to monomeric subunit vaccines, the improved efficacy of Fc- and Fd-mediated oligomeric subunit vaccines is unlikely due to their increased number of neutralizing epitopes because of two reasons. First, in the same mass amounts of monomers and oligomers, the molar amounts of neutralizing epitopes in oligomers are not higher than those in monomers. In fact, because both Fc and Fd tags contain significantly more mass than His tag, His-tagged monomers contain more neutralizing epitopes than Fc- or Fd-tagged oligomers. Second, the current study shows that although both being dimers, JEV EDIII-Fc has significantly higher immunogenicity and protective efficacy than EDIII-EDIII. Then, why compared to monomeric subunit vaccines and loosely packed oligomeric subunit vaccines, tightly packed oligomeric subunit vaccines contain fewer neutralizing epitopes but higher neutralizing immunogenicity and protective efficacy? The reason likely results from fewer non-neutralizing epitopes displayed on the surface of oligomeric subunit vaccines. This can be demonstrated from the tertiary structural models of recombinant EDIII fragments, which shows that recombinant EDIII and EDIII-EDIII contain large artificially exposed surface areas, much of which become re-buried in EDIII-Fc and EDIII-Fd. In summary, Fc- and Fd-mediated oligomerization of viral subunit vaccines can conveniently and effectively overcome the intrinsic limitation of subunit vaccine design by re-burying artificially exposed and non-neutralizing surface regions.

## Data Availability Statement

The original contributions presented in the study are included in the article/supplementary material. Further inquiries can be directed to the corresponding authors.

## Ethics Statement

All experiments involving animals were approved by the Institutional Animal Care and Use Committee (IACUC) of the Laboratory Animal Center, State Key Laboratory of Pathogen and Biosecurity, Beijing Institute of Microbiology and Epidemiology (Permit Number IACUC-DWZX-2021-071).

## Author Contributions

GZ, WT and ML conceived and designed this study. XH, ZC, YD, WT and ML wrote the first draft of the article and finalized it. XH, ZC, YD, XC, HH, YW, YF, GL and MZ performed the experiments, and XH, YW, YZ, BY and WX completed analysis and interpretation the data. GZ and WT make critical comments on the manuscript. GZ, WT and ML revised the article. All authors contributed to the article and approved the submitted version.

## Funding

This work was supported by grants from the National Key Research and Development Plan of China (2021YFC2300200-04), the National Natural Science Foundation of China (32100755), the National Natural Science Foundation of China (82102369), and the Independent Fund of the State Key Laboratory of Pathogen and Biosecurity (SKLPBS2101).

## Conflict of Interest

Authors YD and HH are employed by Beijing Hemu Biotechnology Co., Ltd.

The remaining authors declare that the research was conducted in the absence of any commercial or financial relationships that could be construed as a potential conflict of interest.

## Publisher’s Note

All claims expressed in this article are solely those of the authors and do not necessarily represent those of their affiliated organizations, or those of the publisher, the editors and the reviewers. Any product that may be evaluated in this article, or claim that may be made by its manufacturer, is not guaranteed or endorsed by the publisher.
